# Lipid Oxidation and Barrier Properties of the Coated Freeze-Dried Chicken Meat with Gelatin-Chitosan Film Enriched with Rosemary (*Rosmarinus officinalis* L.) Extract

**DOI:** 10.3390/foods14071127

**Published:** 2025-03-25

**Authors:** Walid Yeddes, Katarzyna Rybak, Iness Bettaieb Rebey, Dorota Pietrzak, Lech Adamczak, Majdi Hammami, Wissem Aidi Wannes, Dorota Witrowa-Rajchert, Moufida Saidani Tounsi, Anne Sylvie Fabiano Tixier, Małgorzata Nowacka

**Affiliations:** 1 Laboratory of Aromatic and Medicinal Plants, Borj Cedria Biotechnology Center, BP901, Hammam-Lif 2050, Tunisia; walid.yeddes@gmail.com (W.Y.); majdi.hammami@cbbc.rnrt.tn (M.H.); aidiwanneswissem18@gmail.com (W.A.W.); moufida.saidani@cbbc.rnrt.tn (M.S.T.); 2 Department of Food Engineering and Process Management, Institute of Food Sciences, Warsaw University of Life Sciences-SGGW, Nowoursynowska 159c, 02-776 Warsaw, Poland; katarzyna_rybak@sggw.edu.pl (K.R.); dorota_witrowa_rajchert@sggw.edu.pl (D.W.-R.); malgorzata_nowacka@sggw.edu.pl (M.N.); 3 Division of Meat Technology, Department of Food Technology, Institute of Food Sciences, Warsaw University of Life Sciences-SGGW, 02-787 Warsaw, Poland; dorota_pietrzak@sggw.edu.pl (D.P.); lech_adamczak@sggw.edu.pl (L.A.); 4 Avignon Université, INRAE, UMR SQPOV, F-84000 Avignon, France

**Keywords:** chicken meat, edible film, rosemary extract, lipid oxidation, barrier properties

## Abstract

The study aimed to evaluate the quality of the coated freeze-dried chicken meat using gelatin-chitosan film enriched with Tunisian rosemary extract. The quality was evaluated on the basis of physical and barrier properties, as well as lipid oxidation for coated and uncoated chicken meat. Chicken breast meat was cut into small pieces, pasteurized, and coated with the active film solution. The active gelatin-chitosan film enriched was prepared with different concentrations of rosemary extract (0 to 2%). The application of the coating solution, with or without ultrasonic treatment was conducted. Subsequently, the samples were subjected to freeze drying for 72 h. The water activity, color, hygroscopic, and rehydration properties of the freeze-dried coated meat were measured. Moreover, the lipid oxidation of the coated vs. uncoated meat was also investigated during a 6 month storage period. Results showed that the coating can form a non-porous surface, which resists the exchange of water vapor, thereby decreasing hygroscopicity and rehydration rates. Likewise, the reduction in the color change and the level of malondialdehyde proved that the active coating allowed good preservation of the meat samples against lipid oxidation during the storage period. However, the application of coating with the use of ultrasound treatment did not positively affect lipid oxidation reduction, and an even higher value was observed in comparison to samples immersed in film forming solutions without ultrasound treatment. It can be concluded that the phenolic compounds incorporated into the film matrix had antioxidant activity, minimizing the extent of lipid oxidation in foods.

## 1. Introduction

In recent decades, the consumption of chicken meat has increased in several nations [1]. Chicken meat, with a low lipid content and low production cost, is a rich source of essential amino acids, unsaturated fatty acids, and minerals for the human body [2]. Its high pH and moisture content make it so that, at aerobic conditions, chicken meat is susceptible to lipid and protein oxidation and microbial growth, leading to a decrease in shelf life [2,3]. The short shelf life of chicken meat is one of the biggest challenges in the retail industry [4]. The shelf life of chicken meat is as short as three to five days in a refrigerator [5]. Hence, the chicken meat industry is interested in extending the shelf life of raw chicken meat. This extended shelf life may be achieved by using an active packaging approach.

Active packaging is regarded as a smart packaging method by incorporating active ingredients, in which a few types of additives/active materials are included within the packaging film or are located at the surface of the packaging containers [6]. It performs an additional function beyond the basic safety functions of traditional packaging methods [7] and comprises interactions among the food products, active substances, and the inner and outer environmental conditions of the packaging materials. The principle of active packaging is based either on the addition of certain ingredients used in a polymer or on particular polymer properties used as the packaging material [2]. The use of active food packaging minimizes hazards and improves the quality and safety of food products [6].

Edible coating is a promising technology of active packaging that includes food, packaging, and preservation in a single concept. This allows for the development of an effective system that preserves the quality of food during the product’s shelf life through the use of biopolymers generated from food industry co-products or underutilized sources of lipids, polysaccharides, or proteins [8]. The edible film made with gelatin and chitosan polymers presents different characteristics, including mechanical properties, thermal stability, light barrier properties, swelling behavior, and water vapor permeability [9]. There is a wide spectrum of natural antioxidants and antimicrobials derived from plants, which have been included as extracts or essential oils in films and coatings [10,11].

Rosemary (*Rosmarinus officinalis* L.) is a perennial aromatic and medicinal plant belonging to the Lamiaceae. Native to the Mediterranean region, rosemary is now widely distributed and has been cultivated around the world [12]. Rosemary has been traditionally used as an antioxidant and food preservative [13]. It is one of the most promising, versatile, and most studied natural preservatives that has been reported to reduce the rate of oxidative reactions and microbial growth in meat products, thereby extending their shelf life. The promising biological and functional characteristics of rosemary extracts are due to the presence of bioactive compounds such as phenolic diterpenes, flavonoids, and triterpenes [14]. Additionally, it has been reported that rosemary extract is effective in delaying lipid oxidation in meat [15,16]. However, its effectiveness when applied to meat packaging has not been extensively investigated. In fact, the purpose of this study was to evaluate the physical properties of the coated freeze-dried chicken meat with gelatin-chitosan film enriched with Tunisian rosemary extract.

## 2. Materials and Methods

### 2.1. Rosemary Extract Characterization

The rosemary extract used in this study was previously characterized by Yeddes et al. [17]. In this section, we describe only its application in the preparation of the gelatin-chitosan film for preserving freeze-dried chicken meat.

Rosemary extract was obtained from Tunisian wild *Rosmarinus officinalis* (Troglodytorum variety) collected from the Chaab El Tweel region (upper-arid bioclimatic zone, 33°47′18.78″ N, 10°13′6.45″ E, 490 m altitude) according to Yeddes et al. [17]. This variety is known for its high content of carnosic acid and rosmarinic acid, contributing to its strong antioxidant properties. Fifteen grams of dried rosemary leaf powder were placed in a Whatman cellulose thimble filter (paper No. 1820-047 grade) in a Soxhlet apparatus and extracted for 3 h with 150 mL of ethanol. Each sample of rosemary extract was stored in brown glass vials with Teflon-sealed caps at 20 ± 0.5 °C [17].

The characterization of the extract revealed significant parameters indicative of its antioxidant properties and chemical composition. Specifically, the total phenolic content was determined to be 31.17 mg of gallic acid equivalents (GA Eq)/g of dry weight (DW), while the total flavonoid content was found to be 2.83 mg of quercetin (Q) Eq/g DW. Furthermore, the carnosic acid content within the extract was measured to be 112.98 mg/g DW, and rosmarinic acid content was determined to be 9.66 mg/g DW. Additionally, the extract exhibited notable antioxidant activity, with a DPPH scavenging activity of 12.72 µg/mL and an ABTS scavenging activity of 72.21 µg/mL. These characterization findings underscore the antioxidant potential and chemical composition of the rosemary extract utilized in the present investigation [17].

### 2.2. Gelatin-Chitosan Film Forming Preparation

A gelatin-chitosan (G-CH) film forming was prepared using a casting procedure based on the method described by Kakaei & Shahbazi [10] with slight adjustments. The chitosan film forming solution was created by dissolving 1% chitosan powder (*w*/*v*) (from shrimp shells (≥75% deacetylated), Sigma–Aldrich, Poznań, Poland)) in a 1% *v*/*v* acetic acid solution and stirring at 1500 rpm for 15 min at room temperature. Similarly, a gelatin film forming solution was made by dissolving 4% gelatin powder from bovine skin (Type B, Sigma-Aldrich, Gillingham, UK) in distilled water, followed by stirring at 1500 rpm for 15 min. The G-CH film forming solution was prepared by combining the gelatin and chitosan solutions in a 50:50 ratio. Glycerol (Sigma–Aldrich, Poznań, Poland) was then added as a plasticizer at a concentration of 75% of the total chitosan and gelatin powder. The entire mixture was stirred using a high-speed homogenizer (IKA Disperseurs T 25 digital ULTRA-TURRAX) at 15,000 rpm for 5 min at 40 °C. A gelatin-chitosan film forming agent containing rosemary ethanolic extract as the active ingredient was prepared using the previously described method. After preparing the G-CH film forming solution as outlined, rosemary ethanolic extract was incorporated into the mixture to achieve final concentrations of 0.5%, 1%, 1.5%, and 2% (*w*/*v*). This addition was followed by homogenization at 15,000 rpm for 3 min and then at 5000 rpm for 30 min at 65 °C to ensure thorough dispersion.

### 2.3. Experimental Design

**Figure 1:**
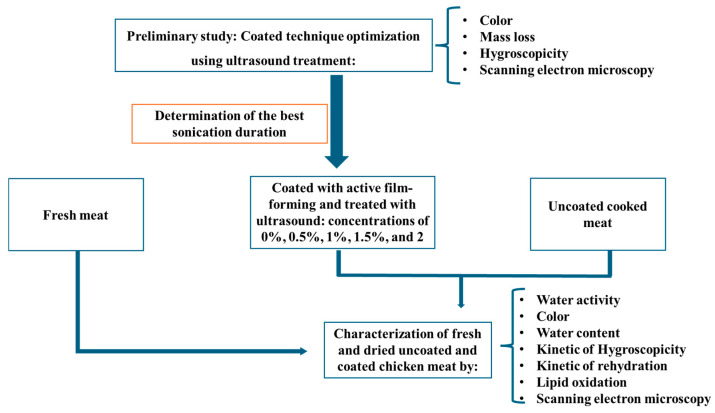
Experimental design.

As shown in Figure 1, this study initially focused on the effect of sonication on chicken meat coating by measuring color, mass loss, hygroscopicity, and performing scanning electron microscopy (SEM). Sonication was applied to improve the homogeneity and adhesion of the gelatin-chitosan film to the chicken meat surface. After optimizing the sonication time for ideal coating, the main objective was to evaluate the impact of adding rosemary extract during the coating process. To achieve this, there were measured water activity, color, water content, the kinetics of hygroscopicity and rehydration, lipid oxidation, and SEM analysis was performed.

### 2.4. Preparation of Chicken Meat Samples

Following the method of Mounir [18], each chicken breast underwent thorough washing with tap water and was trimmed to achieve uniform slices measuring 4 × 1 × 1 cm, with the removal of any fatty parts ensured. These samples were then categorized into seven types of samples, as elaborated in Table 1. The initial batch was designated as the control group. Subsequently, all samples were pasteurized by cooking in hot water at a constant temperature of 88 °C for 5 min. The samples were further divided into 2 groups:

- Control: consisting of uncoated cooked meat
- Coated with active film forming and treated with ultrasound: concentrations of 0%, 0.5%, 1%, 1.5%, and 2%

For each enriched film forming solution, 10 meat samples were immersed in glass beakers and subjected to sonication for 10 min in a 21 kHz ultrasound bath (MKD ULTRASONIC, MKD—3 model, Warsaw, Poland).

### 2.5. Freeze Drying of Meat

All types of samples, including both the control and coated groups, were subjected to freeze drying. For the freeze drying process, 350 g of chicken breast meat was evenly spread on a large horizontal plate with a usable surface area of 989.3 cm^2^ within the drying vessel of a lyophilizer (Christ Gamma 1-16 LSC, Martin Christ Gefriertrocknungsanlagen GmbH, Osterode am Harz, Germany). The pressure within the lyophilizer was maintained at 1.030 mbar, and drying was conducted at a temperature of −40 °C. The freeze drying process lasted for 24 h, resulting in a final water content of 5% on a dry basis (db). Additionally, to assess the kinetics of freeze drying and the water content loss during the process, a smaller sample of 50 g of chicken breast meat was placed on a smaller horizontal plate with a surface area of 176.63 cm^2^. This sample was utilized to monitor the weight loss using the lyophilizer’s integrated balance, which recorded measurements every 15 min over the 24 h drying period. The initial weight of the meat sample was employed to calculate the relative humidity using the following equation:

U_0_ = (M_e_−((M_s_ × M_f_)/100)/((M_s_ × M_f_)/100) (1)

RH = U_0_/U_1_ (2)

where: Me—mass of the sample at a time t;

M_f_—final mass of the sample after total freeze drying;
M_s_—dry matter of the sample after total freeze drying (dried at 105 °C/4 h);
U_0_—moisture content of the chicken meat sample to be freeze-dried at a time t expressed in kg H_2_O/kg d.m.;
U_1_—moisture content of the chicken meat sample to be freeze-dried at a time t + 1 expressed in kg H_2_O/kg d.m.

### 2.6. Assessments and Characterization of Dried Meat Samples

#### 2.6.1. Water Activity

Water activity (a_w_) was determined using the gravimetric method by means of a hygrometer (Test Decagon AquaLab 3TE, METER Group, Uppsala, Sweden). The measurements were conducted in triplicate.

#### 2.6.2. Color

Color analysis of the raw meat, control freeze-dried chicken meat, and coated freeze-dried chicken meat was conducted following the method outlined by Basiak et al. [19], utilizing a colorimeter (Minolta, Model CR-300, Konica Minolta, Tokyo, Japan). The color measurements of the meat samples were performed in triplicate, with the CIE LAB color parameters expressed as L* (lightness), a* (red/green), and b* (yellow/blue).

#### 2.6.3. Water Content (Dry Matter)

The water content (% db dry basis) of raw meat, coated control freeze-dried meat with gelatin-chitosan solution, and coated freeze-dried meat with gelatin-chitosan solution enriched with rosemary extract samples was determined by oven drying at 105 °C for 24 h until a constant weight was reached. The measurement was performed in triplicate [18].

#### 2.6.4. Kinetics of Hygroscopicity (NaCl) (g Water/g Meat)

Approximately 1 g samples of coated freeze-dried chicken meat were placed in desiccators under controlled conditions: 25 °C and 75% relative humidity achieved using a saturated NaCl solution. The weight gain of the samples was monitored at various time intervals over a period of 72 h (0 min, 0.5, 1, 2, 3, 6, 9, 12, 24, 48, and 72 h). Hygroscopicity, representing the amount of water absorbed per 1 g of meat sample, was calculated based on the recorded weight changes. All measurements were conducted in triplicate [20].

#### 2.6.5. Kinetics of Rehydration (in Cold Water)

Coated freeze-dried chicken meat samples treated with gelatin-chitosan solution and enriched with rosemary extract were subjected to a rehydration kinetics study following the methodology outlined by Maurer et al. [21] with slight modifications. Approximately 1 g of meat samples was rehydrated by immersing them in shallow pans containing five liters of water at 25 °C for varying durations ranging from 5 to 30 min (5, 10, 15, 20, and 30 min). The water depth was sufficient to fully submerge the samples. Throughout the kinetic measurement, the meat samples were removed at each time interval and placed on filter paper for one min to remove excess surface moisture. Subsequently, the rehydrated meat samples were weighed, and the rehydration ratio was determined by dividing the initial sample weight (prior to rehydration) by the weight of the sample after rehydration. Additionally, the dry matter content of each tested sample was calculated by drying at 105 °C for 4 h. All measurements were conducted in triplicate to ensure accuracy and reliability.

#### 2.6.6. Kinetics of Rehydration (in Hot Water)

Coated freeze-dried chicken meat samples treated with gelatin-chitosan solution and enriched with rosemary extract underwent rehydration in hot water, following the methodology outlined by Mounir [18], with slight modifications. About 1 g of meat samples was rehydrated by placing them into shallow pans containing five liters of water at 95–100 °C for 3 min. There was always supervision to ensure that the water was deep enough for complete submersion. Meat samples were removed and placed on filter paper for one min. Rehydrated meat samples were weighed, and the rehydration ratio was calculated by dividing the initial sample weight (before rehydration) by the sample weight after rehydration. Dry matter was calculated for each tested sample at 105 °C for 4 h. All measurements were performed in triplicate.

#### 2.6.7. Lipid Oxidation

Lipid oxidation in each coated, freeze-dried chicken meat sample was determined by the spectrophotometric thiobarbituric acid reactive substances (TBARS) test according to the previous method of Chmiel et al. [22]. The extent of lipid oxidation was evaluated by the determining the amount of malonaldehyde (MDA) in samples, which is considered a main marker of oxidative rancidity in meat products. TBARS were measured in duplicate on each sample and expressed as mg malondialdehyde per kg sample (mg MAD/kg).

#### 2.6.8. Scanning Electron Microscopy

Microstructural changes were observed using a scanning electron microscope (SEM) (Hitachi TM3000 Scanning Electron Microscope, Tokyo, Japan). The surface and cross-section microstructure of the treated freeze-dried chicken breast meat samples were evaluated. Samples were placed on a covered stud using carbon adhesive, and the observations were made in a partial vacuum (7 Pa) with an accelerating voltage of 15 kV. Chicken meat samples were observed at a magnification of ×500.

### 2.7. Statistical Analysis

Statistical analysis was performed using IBM SPSS version 15 (IBM Corp., 2016, Armonk, NY, USA). The differences between sample treatments (color differences, water activities, and lipid peroxidation assay) were evaluated using analysis of variance (ANOVA). Duncan’s multiple range tests were employed to compare the means and identify significant differences between groups (*p* < 0.05). All data are presented as mean ± standard deviation (SD).

## 3. Results and Discussion

### 3.1. Optimization of the Coating Process Parameters

**Figure 2:**
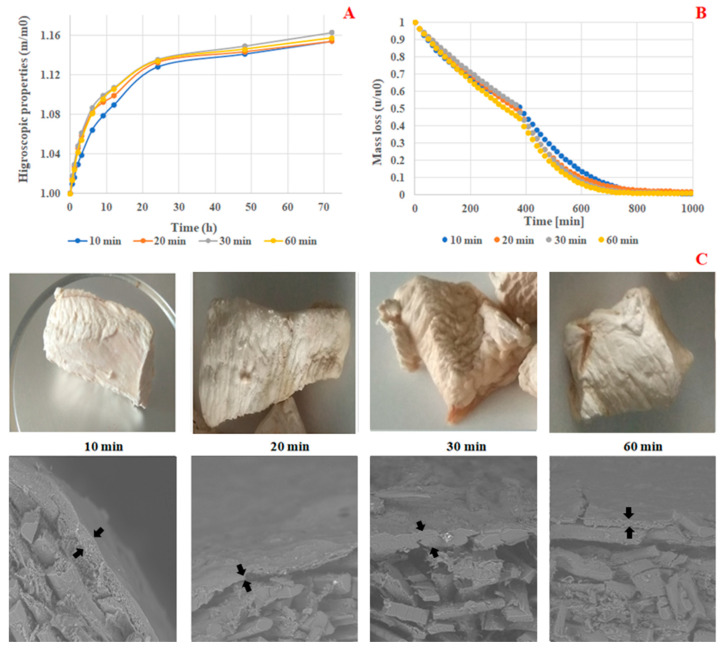
Hygroscopicity kinetics (**A**), relative humidity (**B**), and macro/microstructure, ×500 (**C**), of coated freeze-dried chicken meat treated with ultrasound.

In this section, the ultrasound treatment was evaluated by comparing the cooked meat to the cooked meat treated with ultrasound. The results are summarized in Table 2. The comparison of color difference values (ΔE) between 10 and 60 min demonstrated a significant difference at *p* < 0.05. Specifically, at 10 min, treated meat samples exhibited the lowest ΔE value of 2.756 (Table 2), suggesting that ultrasound treatment minimally impacted color change, with 10 min identified as the optimal processing time. Water activity (aw) measurements similarly showed no significant differences (*p* < 0.05), with treated meat samples at 10 min displaying the lowest aw value of 0.081 (Table 2). Additionally, the hygroscopicity kinetics of coated freeze-dried chicken meat treated for 10 min with ultrasound, as depicted in Figure 2A, demonstrated the most favorable hygroscopic properties. Furthermore, Figure 2B illustrated that coated freeze-dried chicken meat samples treated with ultrasound for 10 min exhibited the highest relative mass loss, which revealed the lowest relative humidity during lyophilization. Overall, the physicochemical characterization of coated freeze-dried chicken breast meat samples treated with an inactive gelatin-chitosan coating during coating process parameter optimization indicated that a treatment duration of 10 min was optimal.

According to Figure 2C, macroscopic observations revealed that chicken meat samples coated with ultrasound treatment applied for 10 min exhibited a better appearance with good shrinkage. Analysis of the microstructure results (cross-section) demonstrated that the thin film layer was well localized on the surface of coated meat samples compared to the uncoated control sample, where the film layer was absent. This indicated the successful coating procedure of the dried chicken meat samples. Similarly, ultrasound treatment applied for 10 min resulted in uniform coating with a homogeneous thick film across the surface of the treated chicken meat, compared to samples coated and subjected to longer treatment durations of 20, 30, and 60 min, which displayed very thin and non-uniform films on the surface of the samples. In summary, it is important to note that the treatment duration of 10 min will be utilized for subsequent experiments throughout the study.

The utilization of ultrasound in meat processing offers a myriad of benefits across various stages of production. Ultrasound treatment has been shown to enhance meat tenderization, facilitate mass transfer processes, improve marination efficiency, aid in freezing, homogenization, crystallization, drying processes, and contribute to microbial inactivation in both poultry and beef [23,24]. In a similar vein, a recent study by (Kim et al. [25] demonstrated the significant benefits of sonication combined with gelatin coating in enhancing the quality of wet-aged pork loins. Their findings revealed that ultrasound treatment increased the moisture content, water retention, and tenderness of the meat while reducing aging and cooking losses. This study further supports our work, which applies ultrasound to bioactive coatings for improving the preservation and quality of chicken meat, highlighting the potential of ultrasound in enhancing both the functional properties of coatings and the overall shelf life of meat products. When combined with sanitizing agents, ultrasound exhibits a synergistic effect on microbial reduction. Studies have demonstrated that high-power ultrasound can notably reduce the Warner-Bratzler shear force and hardness of beef, thereby increasing tenderness without compromising color or elevating drip loss [24,26]. Moreover, ultrasound has been found to enhance the water-holding capacity and tenderness of beef during curing through the induction of protein degradation and modification, as well as the alteration of muscle microstructure [27]. While the application of high-intensity ultrasound to beef pre-rigor may alter ultrastructure and increase calcium release, its effect on the aging rate and final tenderness remains inconclusive [28]. However, low-intensity ultrasound treatment has been observed to have a minimal impact on the shear properties, color stability, and shelf life of vacuum-packaged beef muscles.

### 3.2. Characterization of Chicken Meat Samples Coated with Active Film Forming

#### 3.2.1. Moisture Control

**Figure 3:**
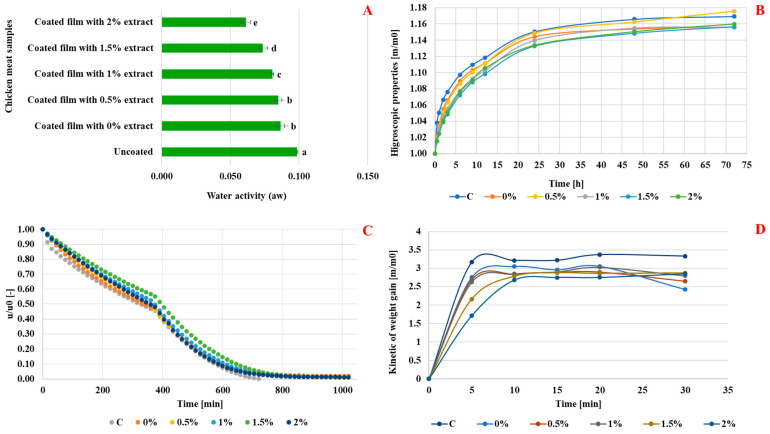
Effect of rosemary enrichment on water activity (**A**), hygroscopic properties (**B**), kinetic of freeze drying (**C**), and kinetic rehydration in cold water (**D**); Values represent the means of triplicate assays ± standard deviation. Different letters (a, b, c, d and e) indicate statistically significant differences (*p* ≤ 0.05).

Analysis of coated meat samples treated with ultrasound (10 min) and dried revealed a gradual decline in relative humidity over time, stabilizing at 800 min (Figure 3C). Compared to uncoated samples, coated samples exhibited slower humidity loss, attributed to the protective barrier of the edible film. Drying kinetics showed two phases: free (0–400 min) and bound (400–1000 min). Beyond 800 min, uncoated meat had significantly lower moisture content, indicating the effectiveness of the active rosemary extract coating in reducing sample permeability. The decrease in humidity can disrupt microorganisms, as drying induces hyperosmotic and oxidative stress, inhibiting microbial activity, aligning with prior research [29].

#### 3.2.2. Evaluation of Color Changes

Table 3 presents the variation in color characteristics of chicken meat coating films with different concentrations of rosemary extract. The parameters measured include L* (lightness), a* (red/green), b* (yellow/blue), hue angle (°hue), and color difference (ΔE). The results indicated that variations in the concentration of rosemary extract in the coating film did not lead to significant differences in the visual appearance of the coated meat samples. This suggests that the addition of rosemary extract did not cause perceptible changes in color that could have been discerned by sensory evaluation. The consistent color characteristics observed across different extract concentrations implied that the coating solution maintained the visual integrity of the meat while incorporating the beneficial properties of rosemary extract. This finding was crucial for ensuring consumer acceptance of coated meat products, as visual appeal played a significant role in consumer perception and purchasing decisions. Overall, these results underscored the suitability of our coating solution for preserving the sensory visual aspect of the meat while potentially enhancing its nutritional and functional properties through the incorporation of rosemary extract. Color is an important factor that affects consumers’ liking for poultry meat [30]. According to Labropoulos et al. [31], this parameter can give an idea about the meat quality and characteristics during the storage period.

#### 3.2.3. Evaluation of Meat Microstructure

Microscopic analysis revealed distinct characteristics in the microstructure of chicken breast meat samples. Uncoated chicken breast meat exhibited a highly porous surface, as depicted in the cross-section (Figure 2C). This porous nature exposes the meat fibers, leaving them unprotected, despite their considerable size, as noted in previous studies by Wattanachant et al. [32]. In contrast, the microstructure of non-active coated samples displayed a non-porous surface with a thick coating layer (Figure 2C), offering protection to the underlying meat fibers. Additionally, chicken breast meat samples coated with active film enriched with increasing concentrations (0.5%, 1%, 1.5%, and 2% *w*/*v*) of rosemary extracts exhibited a non-porous surface and a thick coating layer. This non-porous texture contributed to the samples’ resistance to water vapor exchange, thereby reducing hygroscopicity and the rate of rehydration at room temperature.

The formation of gelatin-chitosan films for potential applications in the food industry, particularly in packaging, has been extensively studied [33]. These films exhibit excellent properties such as degradability, edibility, and film forming capabilities [34]. Incorporating proteins such as gelatin into chitosan films can enhance their structural and functional properties, making them suitable for various applications [35]. Additionally, the use of crosslinking agents such as transglutaminase can further modify the microstructure and properties of these films, potentially improving their mechanical strength and antifungal activities [36]. Overall, the combination of gelatin and chitosan in film formation presents a promising approach for developing sustainable and functional packaging materials with enhanced microstructural characteristics [37]. There is no significant difference between non-active coated samples and chicken breast meat samples coated with active films enriched with increasing concentrations (0.5%, 1%, 1.5%, and 2% *w*/*v*) of rosemary extracts.

#### 3.2.4. Water Activity Measurement

Figure 3A revealed notable differences in water activity levels among the various samples, indicating variations in moisture content and potential shelf stability. Significantly, a significant difference was observed between the water activity of the fresh meat (0.80) and the coated samples. The uncoated meat samples exhibited the highest water activity at 0.099, suggesting maximal moisture availability and a lower potentially extended shelf life due to enhanced microbial growth. Furthermore, as the concentration of rosemary extract increased in the coating film, a decreasing trend in water activity was observed, with values ranging from 0.087 to 0.061. Of particular significance, the coated samples enriched with 2% rosemary extract exhibited the lowest water activity, indicating the most effective moisture control and potential preservation benefits among the coated samples. These findings underscored the impact of the coating film, especially with rosemary extract, on the water activity of the meat samples. The reduced water activity levels in the coated samples suggested improved moisture control and enhanced shelf stability, essential for maintaining product quality and safety during storage and distribution. The freeze drying process played a pivotal role in preserving the meat by minimizing water activity within the matrix, consequently reducing bacterial growth and the risk of germ proliferation. Foschino [38] highlighted that the majority of bacteria thrive in environments with water activity greater than 0.90. Moreover, the layer of active edible film enriched with the extract exhibited significant impermeability to humidity. This enhanced impermeability can be attributed to the increased barrier properties resulting from the grafting of major phenolic compounds of rosemary onto the polymer matrix composed of gelatin and chitosan.

The mechanism of gelatin-chitosan film formation in relation to water activity involves various factors. Gelatin, known for its biocompatibility and film formability [39], when mixed with chitosan, a polymer with antibacterial properties [40], undergoes cross-linking to enhance stability and hydrophilicity [41]. The addition of plasticizers such as glycerol and isosorbide impacts the water uptake and mechanical properties of the composite material [42]. Furthermore, the hydration of chitosan films is influenced by water content, affecting the dielectric and electrical characteristics of the material [43]. Water-responsive behavior in chitosan films is also influenced by the mobility of water molecules, which determines the rate of folding in the material [44]. Overall, the interaction between gelatin and chitosan, along with the presence of plasticizers and water content, plays a crucial role in the film formation mechanism and its response to water activity. Additionally, the incorporation of rosemary extract significantly influences the film’s structure and properties. Thus, the phenolic compounds present in rosemary extract, such as carnosic acid and rosmarinic acid, interact with the polymer matrix, enhancing cross-linking and reducing water permeability. This contributes to improved barrier properties, limiting moisture transfer and reinforcing the film’s ability to protect the coated meat.

#### 3.2.5. Hygroscopicity Kinetic

The hygroscopicity of coated chicken meat samples treated with ultrasound for 10 min and dried by freeze drying is evaluated as a function of time (Figure 3B). The analysis of hygroscopicity results showed that after drying by freeze drying, the samples of chicken meat coated with non-active edible films (0%) treated with ultrasound had a lower hygroscopicity than the control (C). Indeed, the coating based on biopolymers (gelatin-chitosan) formed on the surface of the sample of meat was characterized by a slight impermeability because it may have slightly opposed or delayed the passage of water inside the food matrix. Additionally, the results demonstrated that the hygroscopicity of the chicken meat samples coated with active edible films decreased significantly as the concentration of rosemary extract increased from 0% to 2%.

This significant decrease in moisture permeability is closely related to the barrier property of the film, which has a reduced permeability as a result of the incorporation of certain major phenolic compounds (carnosic acid and rosmarinic acid) in the matrix of polymer (gelatin and chitosan) forming the active edible film. Likewise, the major phenolics compounds of rosemary are grafted onto the functional groups of the polymers cross-linked (gelatin and chitosan) by hydrogen bonds [45,46]. This has led to the reduction of the binding sites of the molecules of water on the surface of the meat coated by active films, and consequently, it allowed reducing the passage of humidity from the external environment into the meat matrix. This phenomenon explains the process of food protection against humidity by the decrease in their hygroscopicity, which consequently leads to an improvement in their duration preservation during storage.

#### 3.2.6. Kinetic Rehydration in Cold Water

According to Figure 3D, this study investigates the rehydration kinetics of chicken meat coated with gelatin-chitosan films enriched with varying concentrations of rosemary extract (0% to 2%) in cold water. The uncoated control sample exhibited the highest rehydration rate of 3.17 after 5 min, attributed to its porous structure allowing rapid water uptake. In contrast, the coated samples, particularly those with higher rosemary extract concentrations, showed progressively lower rehydration rates, with the 2% sample reaching only 1.71 at 5 min. These findings corroborate previous research indicating that edible coatings can effectively reduce moisture absorption [47]. As the rehydration process continued, all samples displayed increased water absorption, yet the control maintained the highest rate (3.37 at 20 min), while the rosemary-coated samples remained significantly lower. Notably, the 1% rosemary extract sample outperformed the 1.5% and 2% samples with a rehydration rate of 3.03 at 20 min, suggesting that a balanced hydrophobic property from rosemary and the coating’s permeability may enhance rehydration efficiency [48]. By the 30-min mark, all samples stabilized in rehydration rates, indicating saturation with ambient water. The coated samples, particularly those with higher rosemary concentrations, demonstrated effective barrier properties that inhibit water penetration, ultimately extending shelf life and reducing spoilage risks [49].

Overall, the results emphasize the potential of gelatin-chitosan coatings enriched with rosemary extract to effectively slow down the rehydration process of freeze-dried chicken meat. Higher concentrations of rosemary significantly diminished water absorption, enhancing the protective function of the coating. The 1% rosemary concentration emerged as optimal, balancing sufficient rehydration with protective benefits against moisture. These findings suggest avenues for further research into optimizing edible coatings to preserve meat products while maintaining their textural qualities, highlighting the role of rosemary’s phenolic compounds in reducing permeability and protecting against microbial growth [21,38,50].

#### 3.2.7. Kinetic Rehydration in Hot Water

**Figure 4:**
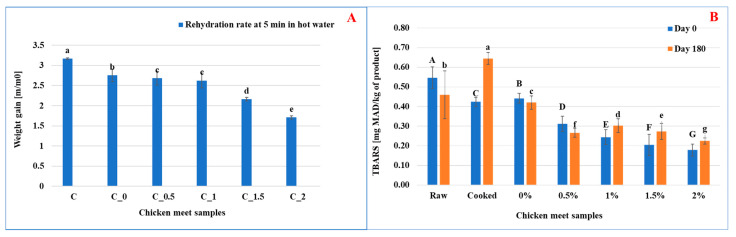
Effect of rosemary enrichment on kinetic rehydration in hot water (**A**), lipid peroxidation index (**B**); Values represent the means of triplicate assays ± standard deviation. Different letters (a, b, c, d, e, f, g and A, B, C, D, E, F, G) indicate statistically significant differences (*p* ≤ 0.05).

According to Figure 4A, the decrease in the rehydration rate in hot water of chicken meat coated with rosemary extract-enriched edible films can be attributed to the hydrophobic properties of rosemary compounds. Rosemary extract contains phenolic compounds such as carnosic acid and carnosol, which are hydrophobic and can form a water-resistant layer on the surface of the film. This barrier limits the ability of water to penetrate the coated meat during the rehydration process in hot water, effectively slowing down water absorption.

The rehydration rate values confirm this trend, showing a progressive decrease with increasing rosemary extract concentration: 3.19 ± 0.02 (Control), 2.8 ± 0.01 (C_0_), 2.68 ± 0.02 (C_0.5_), 2.64 ± 0.02 (C_1_), 2.27 ± 0.01 (C_1.5_), and 1.69 ± 0.01 (C_2_). These results indicate that higher concentrations of rosemary extract result in stronger water resistance, significantly limiting rehydration.

Studies of de Lima et al. [49] suggest that rosemary’s ability to reduce moisture transfer and lipid oxidation enhances its barrier effect.

Additionally, increasing the concentration of rosemary extract in the film results in a thicker and denser coating. A thicker film, with higher solid content from the extract, reduces the film’s porosity and water permeability, making it more difficult for water to pass through during rehydration in hot water. Petersson & Stading [51] reported that edible films with higher concentrations of active compounds tend to have lower water vapor transmission rates, leading to slower water absorption.

The structural properties of the edible film may also change due to crosslinking promoted by bioactive compounds in rosemary. This cross-linking strengthens the film’s matrix, further reducing its water permeability in hot water conditions. Wang et al. [52] found that natural extracts can increase the resistance of edible films to moisture, making them more effective at controlling water uptake during rehydration. Therefore, the combined effects of hydrophobicity, film thickness, and crosslinking explain the reduced rehydration rate in hot water observed in chicken meat coated with films enriched with rosemary extract.

#### 3.2.8. Lipid Oxidation Property

Lipid oxidation is the main process responsible for the quality deterioration of meat and meat products by reducing shelf life [53]. The control, or at least minimization, of the lipid oxidation process is of great interest to the food industry [54]. As shown in Figure 4B, the samples coated with non-active films revealed a slight reduction of the lipid peroxidation index after six months of storage with values of the order of 0.42 ± 0.11 mg MAD/kg of meat compared to the control of 0.44 ± 0.22 mg MAD/kg of meat. This showed that the coating allowed a slight protection of the meat samples against lipid peroxidation. Similarly, this protection has been improved in the case of samples of meat coated with active films enriched with increasing concentrations (0.5, 1, 1.5 to 2% (*w*/*v*)) of ethanolic rosemary extract. The lipid peroxidation index of these samples decreased significantly with the concentration of added extract having 0.26 ± 0.06, 0.30 ± 0.03, 0.27 ± 0.08, and 0.22 ± 0.01 mg MAD/kg of chicken meat. These values were significantly lower than those of the control sample and those of the sample coated with a non-active and dried film and treated with ultrasound, which are of the order of 0.42 ± 0.01 and 0.46 ± 0.03 mg MAD/kg of meat, respectively. In this case, it has been observed that the coating with a film enriched with ethanolic extract of rosemary allowed good preservation of meat samples, chicken against lipid oxidation, for a storage period of 6 months.

Additionally, this proved that the phenolic compounds of rosemary extract have successfully incorporated texture into biopolymer-based film. As previously reported by Yeddes et al. [17], rosemary extract contains a high concentration of phenolic compounds, which contribute to its antioxidant activity. Our findings confirm its efficacy when incorporated into the gelatin-chitosan coating, as evidenced by the reduced lipid oxidation in the treated samples. In this case, it can be concluded that the coating with the active films can represent a new alternative to ensure food preservation against any mediated oxidation by environmental factors during the storage period and can prevent the development of “foul odours” as a result of oxidative rancidity, which constituted a serious problem when storing meat and its products [11]. Similar results were reported in the work of Jridi et al. [55], who reported a reduction in the peroxidation index (TBARS) (level of malondialdehyde (MDA) per g of meat), hence the protective effect against lipid oxidation of beef coated by active gelatin enriched with henna extract (*Lawsonia inermis*) during the conservation.

## 4. Conclusions

This study highlights the effectiveness of gelatin-chitosan films enriched with rosemary extract in enhancing the preservation of freeze-dried chicken meat. The application of this active coating significantly reduced hygroscopicity, limited moisture transfer, and improved the oxidative stability of the meat, thereby extending its shelf life. These benefits are attributed to the synergistic effect of the film matrix and the bioactive compounds of rosemary, which provide a protective barrier against lipid oxidation and environmental factors. Additionally, the incorporation of rosemary extract contributed to the structural integrity of the coating, optimizing its functional properties. These findings suggest that bio-based edible films enriched with plant-derived antioxidants represent a promising alternative for improving the stability and quality of meat products during storage. Future studies could explore sensory analyses with consumers to evaluate the acceptability of these rosemary-enriched films in terms of taste and odor. At higher concentrations, rosemary extract can impart a specific flavor and aroma, which may not be well accepted by all consumers. These tests would have been valuable if incorporated into the current study, as they could offer important insights into consumer preferences. If not performed, these sensory evaluations could represent an essential direction for future research, contributing to the commercial viability of rosemary-based edible coatings in meat preservation. 

## Figures and Tables

**Table 1:** Seven lots of treated meat samples for ulterior use for the freeze drying.

Sample Names	Treatments
Raw	Fresh meat
C	Cooked meat
0%	Coated meat with Ge-CH and treated with US for 10 min
0.5%	Coated meat with 0.5% extract and treated with US for 10 min
1%	Coated meat with 1% extract and treated with US for 10 min
1.5%	Coated meat with 1.5% extract and treated with US for 10 min
2%	Coated meat with 2% extract and treated with US for 10 min

**Table 2:** Evaluation of the color changes of the coated freeze-dried chicken breast meat.

Sonication	10 Min	20 Min	30 Min	60 Min
ΔE	2.756 ^b^ ± 3.916	5.376 ^a^ ± 2.934	7.678 ^a^ ± 3.010	6.269 ^a^ ± 3.522
a_w_	0.081 ^b^ ± 0.0001	0.086 ^b^ ± 0.0004	0.099 ^a^ ± 0.0009	0.087 ^b^ ± 0.0002

Values represent the means of triplicate assays ± standard deviation. Different letters (a, b) indicate statistically significant differences (*p* ≤ 0.05).

**Table 3:** Variation of color characteristics as a function of rosemary extracts added to chicken meat coating film.

	Non-Enrobed	Coating Film with 0% Extract	Coating Film with 0.5% Extract	Coating Film with 1% Extract	Coating Film with 1.5% Extract	Coating Film with 2% Extract
L*	87.77 ^a^ ± 2.7	82.29 ^a^ ±3.12	84.41 ^a^ ± 2.64	84.77 ^a^ ± 2.7	83.23 ^a^ ± 2.55	87.02 ^a^ ± 1.86
a*	−0.67 ^a^ ± 0.5	0.35 ^a^ ± 1.03	−0.21 ^a^ ± 1.08	−1.24 ^a^ ± 0.32	−0.6 ^a^ ± 0.59	−1.34 ^a^ ± 0.27
b*	12.49 ^b^ ± 1.5	17.86 ^a^ ± 1.82	15.15 ^a^ ±2.38	15.55 ^a^ ±2.07	16.09 ^a^ ± 1.86	16.32 ^a^ ± 1.05
°hue	86.75 ^a^ ± 1.9	87.68 ^a^ ± 2.48	87.48 ^a^ ± 1.54	85.35 ^a^ ± 1.28	87.24 ^a^ ± 1.3	85.06 ^a^ ± 0.97
ΔE	Reference	4.66 ^a^ ± 1.43	5.11 ^a^ ± 2.3	4.81 ^a^ ± 2.13	5.7 ^a^ ± 1.25	4.13 ^a^ ± 1.31

Values represent the means of triplicate assays ± standard deviation. Different letters (a, b) indicate statistically significant differences (*p* ≤ 0.05).

## Data Availability

The original contributions presented in the study are included in the article, further inquiries can be directed to the corresponding authors.

## References

[1] Höll L., Behr J., Vogel R.F. (2016). Identification and Growth Dynamics of Meat Spoilage Microorganisms in Modified Atmosphere Packaged Poultry Meat by MALDI-TOF MS. Food Microbiol..

[2] Latou E., Mexis S.F., Badeka A.V., Kontakos S., Kontominas M.G. (2014). Combined Effect of Chitosan and Modified Atmosphere Packaging for Shelf Life Extension of Chicken Breast Fillets. LWT—Food Sci. Technol..

[3] Lorenzo J.M., Sineiro J., Amado I.R., Franco D. (2014). Influence of Natural Extracts on the Shelf Life of Modified Atmosphere-Packaged Pork Patties. Meat Sci..

[4] Petrou S., Tsiraki M., Giatrakou V., Savvaidis I.N. (2012). Chitosan Dipping or Oregano Oil Treatments, Singly or Combined on Modified Atmosphere Packaged Chicken Breast Meat. Int. J. Food Microbiol..

[5] Mojaddar Langroodi A., Tajik H., Mehdizadeh T., Moradi M., Moghaddas Kia E., Mahmoudian A. (2018). Effects of Sumac Extract Dipping and Chitosan Coating Enriched with Zataria Multiflora Boiss Oil on the Shelf-Life of Meat in Modified atmosphere Packaging. LWT—Food Sci. Technol..

[6] Ahmed I., Lin H., Zou L., Brody A.L., Li Z., Qazi I.M., Pavase T.R., Lv L. (2017). A Comprehensive Review on the Application of Active Packaging Technologies to Muscle Foods. Food Control.

[7] Bastarrachea L.J., Wong D.E., Roman M.J., Lin Z., Goddard J.M. (2015). Active Packaging Coatings. Coatings.

[8] Umaraw P., Munekata P.E.S., Verma A.K., Barba F.J., Singh V.P., Kumar P., Lorenzo J.M. (2020). Edible Films/Coating with Tailored Properties for Active Packaging of Meat, Fish and Derived Products. Trends Food Sci. Technol..

[9] Kakaei S., Shahbazi Y. (2016). Effect of Chitosan-Gelatin Film Incorporated with Ethanolic Red Grape Seed Extract and Ziziphora Clinopodioides Essential Oil on Survival of Listeria Monocytogenes and Chemical, Microbial and Sensory Properties of Minced Trout Fillet. LWT—Food Sci. Technol..

[10] Domínguez R., Barba F.J., Gómez B., Putnik P., Bursać Kovačević D., Pateiro M., Santos E.M., Lorenzo J.M. (2018). Active Packaging Films with Natural Antioxidants to Be Used in Meat Industry: A Review. Food Res. Int..

[11] Lorenzo J.M., Batlle R., Gómez M. (2014). Extension of the Shelf-Life of Foal Meat with Two Antioxidant Active Packaging Systems. LWT—Food Sci. Technol..

[12] Hamidpour R., Elias G., Hamidpour S. (2017). *Rosmarinus officinalis* (Rosemary): A Novel Therapeutic Agent for Antioxidant, Antimicrobial, Anticancer, Antidiabetic, Antidepressant, Neuroprotective, AntiInflammatory, and Anti-obesity Treatment. Biomed. J. Sci. Tech. Res..

[13] Couto R.O., Conceição E.C., Chaul L.T., Oliveira E.M.S., Martins F.S., Bara M.T.F., Rezende K.R., Alves S.F., Paula J.R. (2012). Spray-Dried Rosemary Extracts: Physicochemical and Antioxidant Properties. Food Chem..

[14] Kaur R., Gupta T.B., Bronlund J., Kaur L. (2023). The Potential of Rosemary as a Functional Ingredient for Meat Products—A Review. Food Rev. Int..

[15] Georgantelis D., Ambrosiadis I., Katikou P., Blekas G., Georgakis S.A. (2007). Effect of Rosemary Extract, Chitosan and α-Tocopherol on Microbiological Parameters and Lipid Oxidation of Fresh Pork Sausages Stored at 4 °C. Meat Sci..

[16] Kumar Y., Yadav D.N., Ahmad T., Narsaiah K. (2015). Recent Trends in the Use of Natural Antioxidants for Meat and Meat Products. Compr. Rev. Food Sci. Food Saf..

[17] Yeddes W., Chalghoum A., Aidi-Wannes W., Ksouri R., Saidani Tounsi M. (2019). Effect of Bioclimatic Area and Season on Phenolics and Antioxidant Activities of Rosemary (*Rosmarinus officinalis* L.) Leaves. J. Essent. Oil Res..

[18] Mounir S. (2015). Texturing of Chicken Breast Meat as an Innovative Way to Intensify Drying: Use of a Coupled Washing/Diffusion CWD Phenomenological Model to Enhance Kinetics and Functional Properties. Dry. Technol..

[19] Basiak E., Lenart A., Debeaufort F. (2018). How Glycerol and Water Contents Affect the Structural and Functional Properties of Starch-Based Edible Films. Polymers.

[20] Samborska K., Bieńkowska B. (2013). Physicochemical Properties of Spray Dried Honey Preparations. Adv. Agric. Sci. Probl. Issues.

[21] Maurer A.J., Baker R.C., Vadehra D.V. (1973). Factors Affecting the Drying, Stability, and Rehydration of Freeze-Dried Chicken Meat. Poult. Sci..

[22] Chmiel M., Roszko M., Adamczak L., Florowski T., Pietrzak D. (2019). Influence of Storage and Packaging Method on Chicken Breast Meat Chemical Composition and Fat Oxidation. Poult. Sci..

[23] Al-Hilphy A.R., Al-Temimi A.B., Al Rubaiy H.H.M., Anand U., Delgado-Pando G., Lakhssassi N. (2020). Ultrasound Applications in Poultry Meat Processing: A Systematic Review. J. Food Sci..

[24] Jayasooriya S.D., Torley P.J., D’Arcy B.R., Bhandari B.R. (2007). Effect of High Power Ultrasound and Ageing on the Physical Properties of Bovine *Semitendinosus* and *Longissimus* Muscles. Meat Sci..

[25] Kim Y.-J., Jung T.-J., Kim T.-K., Lee J.H., Shin D.-M., Yu H.H., Choi Y.-S. (2023). The Effect of Gelatin Coating and Sonication on the Quality Properties of Wet-Aging Pork Loins. Food Sci. Anim. Resour..

[26] Kang D., Gao X., Ge Q., Zhou G., Zhang W. (2017). Effects of Ultrasound on the Beef Structure and Water Distribution during Curing through Protein Degradation and Modification. Ultrason. Sonochem..

[27] Li K., Kang Z.-L., Zhao Y.-Y., Xu X.-L., Zhou G.-H. (2014). Use of High-Intensity Ultrasound to Improve Functional Properties of Batter Suspensions Prepared from PSE-like Chicken Breast Meat. Food Bioprocess Technol..

[28] Got F., Culioli J., Berge P., Vignon X., Astruc T., Quideau J.M., Lethiecq M. (1999). Effects of High-Intensity High-Frequency Ultrasound on Ageing Rate, Ultrastructure and Some Physico-Chemical Properties of Beef. Meat Sci..

[29] Jay J.M., Loessner M.J., Golden D.A. (2005). Protection of Foods by Drying. Modern Food Microbiology.

[30] Wideman N., O’Bryan C., Crandall P. (2016). Factors Affecting Poultry Meat Colour and Consumer Preferences—A Review. Worlds Poult. Sci. J..

[31] Labropoulos A.E., Varzakas T., Anestis S., Kostas T., Panagiotou P. (2013). Preparation, Storage and Distribution of Coated and Uncoated Chicken Meat Products. Int. J. Food Eng..

[32] Wattanachant S., Benjakul S., Ledward D.A. (2005). Microstructure and Thermal Characteristics of Thai Indigenous and Broiler Chicken Muscles. Poult. Sci..

[33] Guo L., Wang Y.-J., Ren L., Leng Y.-X., Gao Q.-Y., Ge J. (2008). Preparation and Characterization of Gelatin-Immobilized Chitosan Film. J. Funct. Mater..

[34] Mallmann E., Maia F., Mazzetto S., Fechine P. (2011). Microstructure and Magneto-Dielectric Properties of the Chitosan/Gelatin-YIG Biocomposites. Express Polym. Lett..

[35] Priyadarshi R., Rhim J.-W. (2020). Chitosan-Based Biodegradable Functional Films for Food Packaging Applications. Innov. Food Sci. Emerg. Technol..

[36] Bialik-Wąs K., Królicka E., Malina D. (2021). Impact of the Type of Crosslinking Agents on the Properties of Modified Sodium Alginate/Poly(Vinyl Alcohol) Hydrogels. Molecules.

[37] Cai L., Shi H., Cao A., Jia J. (2019). Characterization of Gelatin/Chitosan Ploymer Films Integrated with Docosahexaenoic Acids Fabricated by Different Methods. Sci. Rep..

[38] Foschino R. (2006). James M. Jay, Martin J. Loessner, David A. Golden Modern food microbiology. Ann. Microbiol..

[39] Duan Q., Chen Y., Yu L., Xie F. (2022). Chitosan–Gelatin Films: Plasticizers/Nanofillers Affect Chain Interactions and Material Properties in Different Ways. Polymers.

[40] Mathesan S., Rath A., Ghosh P. (2017). Insights on Water Dynamics in the Hygromorphic Phenomenon of Biopolymer Films. J. Phys. Chem. B.

[41] Murugaraj P., Mainwaring D.E., Tonkin D.C., Al Kobaisi M. (2011). Probing the Dynamics of Water in Chitosan Polymer Films by Dielectric Spectroscopy. J. Appl. Polym. Sci..

[42] Umar A.K., Sriwidodo S., Maksum I.P., Wathoni N. (2021). Film-Forming Spray of Water-Soluble Chitosan Containing Liposome-Coated Human Epidermal Growth Factor for Wound Healing. Molecules.

[43] Lou C.W., Hu J.J., Lu C.T., Huang C.C., Huang M.S., Lin J.H. (2011). Manufacturing of Functional Gelatin/Chitosan Composite Membrane. Adv. Mater. Res..

[44] Wiles J.l., Vergano P.j., Barron F.h., Bunn J.m., Testin R.f. (2000). Water Vapor Transmission Rates and Sorption Behavior of Chitosan Films. J. Food Sci..

[45] Hoque M.d.S., Benjakul S., Prodpran T. (2011). Properties of Film from Cuttlefish (Sepia Pharaonis) Skin Gelatin Incorporated with Cinnamon, Clove and Star Anise Extracts. Food Hydrocoll..

[46] Siripatrawan U., Harte B. (2010). Physical Properties and Antioxidant Activity of a Journal of the Science of Food and Agriculture n Active Film from Chitosan Incorporated with Green Tea Extract. Food Hydrocoll..

[47] Yazicioglu N., Mert I., Özmen T., Öztürk Ş., Saritaş E., Özer R. (2025). Development of Edible Coating Incorporating Cherry Stem Powder or Leek Powder to Decrease Oil Uptake and Lipid Oxidation in Potatoes During Air, Oven, and Deep Oil Frying Methods. Potato Res..

[48] Khin M.M., Zhou W., Perera C.O. (2006). A Study of the Mass Transfer in Osmotic Dehydration of Coated Potato Cubes. J. Food Eng..

[49] de Lima A.F., Leite R.H.d.L., Pereira M.W.F., Silva M.R.L., de Araújo T.L.A.C., de Lima Júnior D.M., Gomes M.d.N.B., Lima P.d.O. (2024). Chitosan Coating with Rosemary Extract Increases Shelf Life and Reduces Water Losses from Beef. Foods.

[50] Farkas B.e., Singh R.p. (1991). Physical Properties of Air-Dried and Freeze-Dried Chicken White Meat. J. Food Sci..

[51] Petersson M., Stading M. (2005). Water Vapour Permeability and Mechanical Properties of Mixed Starch-Monoglyceride Films and Effect of Film Forming Conditions. Food Hydrocoll..

[52] Wang Y., McClements D.J., Peng X., Xu Z., Meng M., Ji H., Zhi C., Ye L., Zhao J., Jin Z. (2024). Effects of Crosslinking Agents on Properties of Starch-Based Intelligent Labels for Food Freshness Detection. Int. J. Biol. Macromol..

[53] Min B., Ahn D.U. (2005). Mechanism of Lipid Peroxidation in Meat and Meat Products—A Review. Food Sci. Biotechnol..

[54] Brøndum J., Byrne D.V., Bak L.S., Bertelsen G., Engelsen S.B. (2000). Warmed-over Flavour in Porcine Meat—A Combined Spectroscopic, Sensory and Chemometric Study. Meat Sci..

[55] Jridi M., Mora L., Souissi N., Aristoy M.-C., Toldrá F. (2017). Effects of Active Gelatin Coated with Henna (*L. Inermis*) Extract on Beef Meat Quality during Chilled Storage. Food Control.

